# Longitudinal detection of astroviruses in feral pigeon droppings reveals a year-round source of viral exposure for endemic bats

**DOI:** 10.1128/jvi.00657-26

**Published:** 2026-07-16

**Authors:** Léane Le Bodic, Maëliss Hoarau, Kiliann Heinrich, Muriel Dietrich, Camille Lebarbenchon

**Affiliations:** 1 Université de La Réunion, UMR Processus Infectieux en Milieu Insulaire Tropical, Inserm, CNRS, IRD, Saint Denis de La Réunion, France; Emory University School of Medicine, Atlanta, Georgia, USA

**Keywords:** *Astroviridae*, Southwestern Indian Ocean, *Columba livia*, *Mormopterus francoismoutoui*, Reunion Island

## LETTER

Astroviruses (AstVs; family *Astroviridae*) are widespread and highly diverse viruses infecting a broad range of mammalian and avian hosts, including humans (1, 2). They are associated with diseases ranging from gastroenteritis and nephritis to neurological disorders. While classified into distinct mammalian (*Mamastrovirus*) and avian (*Avastrovirus*) genera, their evolutionary history reveals frequent cross-species transmission, indicating low host specificity. This suggests a high capacity for circulation among sympatric vertebrate hosts sharing habitats or ecosystems (3). However, the epidemiological and pathological consequences of such interspecies transmissions, in particular between avian and mammalian hosts, remain poorly understood.

In a previous study, we reported high astrovirus (AstV) diversity in an endemic bat species (*Mormopterus francoismoutoui*) on Réunion Island, suggesting repeated spillover from synanthropic rodents and birds (4). Here, we investigated the persistence and genetic diversity of AstVs in feral pigeons (*Columba livia*). Our objective was to determine whether bat exposure to pigeon AstVs is episodic and limited to specific viral variants or could represent a continuous source of genetically diverse viruses.

Fresh pigeon droppings were collected from the ground and around nests at a shared roosting site (urban bridge in Sainte-Marie; −20.90°S, 55.55°E), between feral pigeons and the endemic bat *M. francoismoutoui*. Sampling was conducted between November 2024 and November 2025 (32 sampling sessions; 2–20 samples/session, mean = 9). Samples were collected using sterile cotton-tipped applicators and placed in 1 mL of Minimum Essential Medium. Viral RNA extraction, cDNA synthesis (4), RNA-dependent RNA polymerase amplification by semi-nested PCR (the expected product size of the second PCR was 422 bp; 5), and Sanger sequencing (Genoscreen, Lille, France) were performed. Phylogenetic analysis was performed with pigeon and bat AstV sequences from our previous studies (4), together with reference sequences, with PhyML 3.1 (6).

Astroviruses were detected in 21% of samples (58/281). All PCR-positive samples were confirmed by sequencing. Positive samples were identified in 12 of the 13 months of the study period (Table S1, Fig. S1), although detection varied significantly among months (*χ*² =41, df = 12, *P* < 0.001). While our environmental sampling design and limited sample sizes per session preclude robust estimation of infection prevalence in pigeons, these results indicate year-round circulation, with an overall proportion of positive samples comparable to that reported in *Rattus rattus* on Reunion Island (19%; 4).

**Fig 1:**
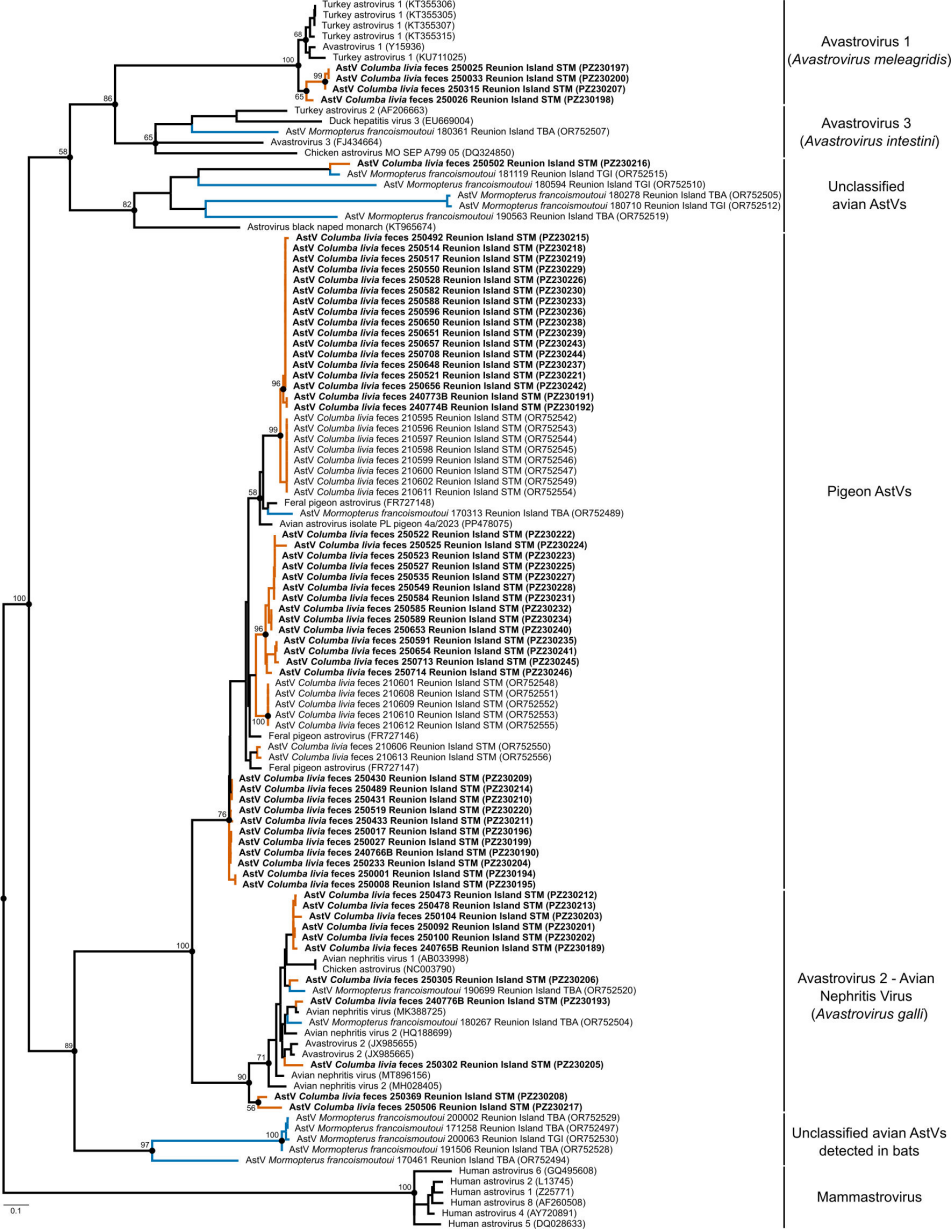
Maximum likelihood consensus tree inferred from 116 astrovirus (AstV) RNA-dependent RNA polymerase partial nucleotide sequences (397 bp). The tree was generated using a general time-reversible model with gamma-distributed rate variation among sites (α = 0.8) and a proportion of invariant sites (*I* = 0.08). Bootstrap support values are shown for external nodes (1,000 replicates, non-parametric standard bootstrap). Astrovirus sequences obtained from feral pigeons (*Columba livia*) on Reunion Island are highlighted in orange, while those detected in the endemic bat species *Mormopterus francoismoutoui* are shown in blue (from a previous study; 4). Accession numbers in bold indicate sequences generated in this study.

Pairwise sequence identity ranged from 49% to 100%, highlighting substantial genetic diversity. Eleven sequences clustered with Avian Nephritis Virus (Avastrovirus 2/*Avastrovirus galli*; Fig. 1), and 42 formed a closely related yet distinct “Pigeon AstV clade,” consistent with previous reports (7–10). Pairwise sequence identity between these two clades ranged from 67% to 74%. Four sequences were phylogenetically related to Turkey astrovirus 1 (Avastrovirus 1/*A. meleagridis*; Fig. 1). One sequence was in the unclassified Avastrovirus group, with a pairwise sequence identity to other viruses ranging from 49% to 55%. None of the AstVs sequences detected in this study clustered with Turkey astrovirus 2 sequences (Avastrovirus 3/*A. intestini*).

To our knowledge, this is the first longitudinal study demonstrating that feral pigeons constitute a continuous source of genetically diverse AstVs. Results highlight the role of synanthropic species in shaping viral exposure dynamics in endemic wildlife on tropical islands. Further investigations are warranted, as the pathological and ecological consequences of avian-to-mammalian AstV spillovers remain unknown.

## Data Availability

Data are available in Table S1 and in GenBank under accession numbers PZ230189 to PZ230246.

## SUPPLEMENTAL MATERIAL

Figure S1 jvi.00657-26-s0001.tiff Astrovirus detection in feral pigeon droppings across 32 sampling sessions.

Supplemental legends jvi.00657-26-s0002.docx Descriptive legends for Table S1 and Fig. S1.

Table S1 jvi.00657-26-s0003.pdf Sample information and accession numbers of sequences generated in this study.

ASM does not own the copyrights to Supplemental Material that may be linked to, or accessed through, an article. The authors have granted ASM a non-exclusive, world-wide license to publish the Supplemental Material files. Please contact the corresponding author directly for reuse.

## References

[1] De Benedictis P, Schultz-Cherry S, Burnham A, Cattoli G. 2011. Astrovirus infections in humans and animals – molecular biology, genetic diversity, and interspecies transmissions. Infect Genet Evol 11:1529–1544. doi:10.1016/j.meegid.2011.07.024 21843659 PMC7185765

[2] Bosch A, Pintó RM, Guix S. 2014. Human astroviruses. Clin Microbiol Rev 27:1048–1074. doi:10.1128/CMR.00013-14 25278582 PMC4187635

[3] Mendenhall IH, Smith GJD, Vijaykrishna D. 2015. Ecological drivers of virus evolution: astrovirus as a case study. J Virol 89:6978–6981. doi:10.1128/JVI.02971-14 25948751 PMC4473558

[4] Leong R, Hoarau AOG, Carcauzon V, Köster M, Dietrich M, Tortosa P, Lebarbenchon C. 2025. High astrovirus diversity in an endemic bat species suggests multiple spillovers from synanthropic rodents and birds. J Virol 99:e0135724. doi:10.1128/jvi.01357-24 39840948 PMC11853114

[5] Chu DKW, Poon LLM, Guan Y, Peiris JSM. 2008. Novel astroviruses in insectivorous bats. J Virol 82:9107–9114. doi:10.1128/JVI.00857-08 18550669 PMC2546893

[6] Guindon S, Dufayard J-F, Lefort V, Anisimova M, Hordijk W, Gascuel O. 2010. New algorithms and methods to estimate maximum-likelihood phylogenies: assessing the performance of PhyML 3.0. Syst Biol 59:307–321. doi:10.1093/sysbio/syq010 20525638

[7] Zhao W, Zhu AL, Yu Y, Yuan CL, Zhu CX, Yang ZB, Cui L, Hua XG. 2011. Complete sequence and genetic characterization of pigeon avian nephritis virus, a member of the family Astroviridae. Arch Virol 156:1559–1565. doi:10.1007/s00705-011-1034-8 21618030

[8] Łukaszuk E, Dziewulska D, Custer JM, Kraberger S, Varsani A, Stenzel T. 2024. Occurrence of astrovirus in young racing pigeons and genome characterization of 2 new astrovirus genomes representing 2 new species. Poult Sci 103:104028. doi:10.1016/j.psj.2024.104028 39043026 PMC11318551

[9] Zhao W, Zhu AL, Yuan CL, Yu Y, Zhu CX, Lan DL, Yang ZB, Cui L, Hua XG. 2011. Detection of astrovirus infection in pigeons (*Columbia livia*) during an outbreak of diarrhoea. Avian Pathol 40:361–365. doi:10.1080/03079457.2011.587792 21812714

[10] Kofstad T, Jonassen CM. 2011. Screening of feral and wood pigeons for viruses harbouring a conserved mobile viral element: characterization of novel astroviruses and picornaviruses. PLoS One 6:e25964. doi:10.1371/journal.pone.0025964 22043297 PMC3197151

