# Supplementary material for: Longitudinal detection of astroviruses in feral pigeon droppings reveals a year-round source of viral exposure for endemic bats
Source: J Virol. 2026 Jul 16;100(8):e00657-26. doi: 10.1128/jvi.00657-26 (PMC13483236; doi:10.1128/jvi.00657-26)
Supplement: Supplementary file 1 [file jvi.00657-26-s0003.pdf]

Sample	Host species	Sampling date	Astrovirus PCR positive	Genbank accession number	Astrovirus type
24_0753B	*Columba livia*	29/11/2024	0		
24_0754B	*Columba livia*	29/11/2024	0		
24_0755B	*Columba livia*	29/11/2024	0		
24_0756B	*Columba livia*	29/11/2024	0		
24_0757B	*Columba livia*	29/11/2024	0		
24_0758B	*Columba livia*	29/11/2024	0		
24_0759B	*Columba livia*	29/11/2024	0		
24_0760B	*Columba livia*	29/11/2024	0		
24_0761B	*Columba livia*	29/11/2024	0		
24_0762B	*Columba livia*	29/11/2024	0		
24_0763B	*Columba livia*	29/11/2024	0		
24_0764B	*Columba livia*	29/11/2024	0		
24_0765B	*Columba livia*	29/11/2024	1	PZ230189	Avastrovirus 2
24_0766B	*Columba livia*	29/11/2024	1	PZ230190	Pigeon AstVs
24_0767B	*Columba livia*	10/12/2024	0		
24_0768B	*Columba livia*	10/12/2024	0		
24_0769B	*Columba livia*	10/12/2024	0		
24_0770B	*Columba livia*	10/12/2024	0		
24_0771B	*Columba livia*	10/12/2024	0		
24_0772B	*Columba livia*	10/12/2024	0		
24_0773B	*Columba livia*	10/12/2024	1	PZ230191	Pigeon AstVs
24_0774B	*Columba livia*	10/12/2024	1	PZ230192	Pigeon AstVs
24_0775B	*Columba livia*	10/12/2024	0		
24_0776B	*Columba livia*	10/12/2024	1	PZ230193	Avastrovirus 2
24_0777B	*Columba livia*	10/12/2024	0		
24_0778B	*Columba livia*	10/12/2024	0		
24_0779B	*Columba livia*	10/12/2024	0		
24_0780B	*Columba livia*	10/12/2024	0		
25_001	*Columba livia*	09/01/2025	1	PZ230194	Pigeon AstVs
25_002	*Columba livia*	09/01/2025	0		
25_003	*Columba livia*	09/01/2025	0		
25_004	*Columba livia*	09/01/2025	0		
25_005	*Columba livia*	09/01/2025	0		
25_006	*Columba livia*	09/01/2025	0		
25_007	*Columba livia*	09/01/2025	0		
25_008	*Columba livia*	09/01/2025	1	PZ230195	Pigeon AstVs
25_009	*Columba livia*	09/01/2025	0		
25_010	*Columba livia*	09/01/2025	0		
25_011	*Columba livia*	09/01/2025	0		
25_012	*Columba livia*	09/01/2025	0		
25_013	*Columba livia*	09/01/2025	0		
25_014	*Columba livia*	09/01/2025	0		
25_015	*Columba livia*	20/01/2025	0		
25_016	*Columba livia*	20/01/2025	0		
25_017	*Columba livia*	20/01/2025	1	PZ230196	Pigeon AstVs
25_018	*Columba livia*	20/01/2025	0		
25_019	*Columba livia*	20/01/2025	0		
25_020	*Columba livia*	20/01/2025	0		
25_021	*Columba livia*	20/01/2025	0		
25_022	*Columba livia*	20/01/2025	0		
25_023	*Columba livia*	20/01/2025	0		
25_024	*Columba livia*	20/01/2025	0		
25_025	*Columba livia*	20/01/2025	1	PZ230197	Avastrovirus 1
25_026	*Columba livia*	20/01/2025	1	PZ230198	Avastrovirus 1
25_027	*Columba livia*	20/01/2025	1	PZ230199	Pigeon AstVs
25_028	*Columba livia*	20/01/2025	0		
25_029	*Columba livia*	20/01/2025	0		
25_030	*Columba livia*	20/01/2025	0		
25_031	*Columba livia*	20/01/2025	0		
25_032	*Columba livia*	20/01/2025	0		
25_033	*Columba livia*	20/01/2025	1	PZ230200	Avastrovirus 1
25_034	*Columba livia*	20/01/2025	0		
25_085	*Columba livia*	31/01/2025	0		
25_086	*Columba livia*	31/01/2025	0		
25_087	*Columba livia*	31/01/2025	0		
25_088	*Columba livia*	31/01/2025	0		
25_089	*Columba livia*	31/01/2025	0		
25_090	*Columba livia*	31/01/2025	0		
25_091	*Columba livia*	10/02/2025	0		
25_092	*Columba livia*	10/02/2025	1	PZ230201	Avastrovirus 2
25_093	*Columba livia*	10/02/2025	0		
25_094	*Columba livia*	10/02/2025	0		
25_095	*Columba livia*	10/02/2025	0		
25_096	*Columba livia*	10/02/2025	0		
25_097	*Columba livia*	10/02/2025	0		
25_098	*Columba livia*	10/02/2025	0		
25_099	*Columba livia*	10/02/2025	0		

25_100	*Columba livia*	10/02/2025	1	PZ230202	Avastrovirus 2
25_101	*Columba livia*	10/02/2025	0		
25_102	*Columba livia*	10/02/2025	0		
25_103	*Columba livia*	10/02/2025	0		
25_104	*Columba livia*	10/02/2025	1	PZ230203	Avastrovirus 2
25_155	*Columba livia*	20/02/2025	0		
25_156	*Columba livia*	20/02/2025	0		
25_157	*Columba livia*	20/02/2025	0		
25_158	*Columba livia*	20/02/2025	0		
25_159	*Columba livia*	20/02/2025	0		
25_160	*Columba livia*	20/02/2025	0		
25_161	*Columba livia*	20/02/2025	0		
25_162	*Columba livia*	20/02/2025	0		
25_163	*Columba livia*	20/02/2025	0		
25_164	*Columba livia*	20/02/2025	0		
25_165	*Columba livia*	20/02/2025	0		
25_166	*Columba livia*	20/02/2025	0		
25_167	*Columba livia*	03/03/2025	0		
25_168	*Columba livia*	03/03/2025	0		
25_169	*Columba livia*	03/03/2025	0		
25_170	*Columba livia*	03/03/2025	0		
25_171	*Columba livia*	03/03/2025	0		
25_172	*Columba livia*	03/03/2025	0		
25_173	*Columba livia*	03/03/2025	0		
25_174	*Columba livia*	03/03/2025	0		
25_175	*Columba livia*	03/03/2025	0		
25_176	*Columba livia*	03/03/2025	0		
25_177	*Columba livia*	03/03/2025	0		
25_178	*Columba livia*	03/03/2025	0		
25_230	*Columba livia*	14/03/2025	0		
25_231	*Columba livia*	14/03/2025	0		
25_232	*Columba livia*	14/03/2025	0		
25_233	*Columba livia*	14/03/2025	1	PZ230204	Pigeon AstVs
25_234	*Columba livia*	14/03/2025	0		
25_235	*Columba livia*	14/03/2025	0		
25_236	*Columba livia*	24/03/2025	0		
25_237	*Columba livia*	24/03/2025	0		
25_238	*Columba livia*	24/03/2025	0		
25_290	*Columba livia*	31/03/2025	0		
25_291	*Columba livia*	31/03/2025	0		
25_292	*Columba livia*	31/03/2025	0		
25_293	*Columba livia*	31/03/2025	0		
25_294	*Columba livia*	07/04/2025	0		
25_295	*Columba livia*	07/04/2025	0		
25_296	*Columba livia*	07/04/2025	0		
25_297	*Columba livia*	07/04/2025	0		
25_298	*Columba livia*	07/04/2025	0		
25_299	*Columba livia*	07/04/2025	0		
25_300	*Columba livia*	07/04/2025	0		
25_301	*Columba livia*	07/04/2025	0		
25_302	*Columba livia*	07/04/2025	1	PZ230205	Avastrovirus 2
25_303	*Columba livia*	07/04/2025	0		
25_304	*Columba livia*	07/04/2025	0		
25_305	*Columba livia*	07/04/2025	1	PZ230206	Avastrovirus 2
25_306	*Columba livia*	07/04/2025	0		
25_307	*Columba livia*	07/04/2025	0		
25_308	*Columba livia*	07/04/2025	0		
25_309	*Columba livia*	14/04/2025	0		
25_310	*Columba livia*	14/04/2025	0		
25_311	*Columba livia*	14/04/2025	0		
25_312	*Columba livia*	14/04/2025	0		
25_313	*Columba livia*	14/04/2025	0		
25_314	*Columba livia*	14/04/2025	0		
25_315	*Columba livia*	14/04/2025	1	PZ230207	Avastrovirus 1
25_364	*Columba livia*	22/04/2025	0		
25_365	*Columba livia*	22/04/2025	0		
25_366	*Columba livia*	22/04/2025	0		
25_367	*Columba livia*	22/04/2025	0		
25_368	*Columba livia*	22/04/2025	0		
25_369	*Columba livia*	22/04/2025	1	PZ230208	Avastrovirus 2
25_370	*Columba livia*	22/04/2025	0		
25_371	*Columba livia*	22/04/2025	0		
25_372	*Columba livia*	22/04/2025	0		
25_373	*Columba livia*	28/04/2025	0		
25_374	*Columba livia*	28/04/2025	0		
25_375	*Columba livia*	28/04/2025	0		
25_376	*Columba livia*	28/04/2025	0		
25_377	*Columba livia*	28/04/2025	0		

25_378	*Columba livia*	05/05/2025	0		
25_379	*Columba livia*	05/05/2025	0		
25_428	*Columba livia*	12/05/2025	0		
25_429	*Columba livia*	12/05/2025	0		
25_430	*Columba livia*	19/05/2025	1	PZ230209	Pigeon AstVs
25_431	*Columba livia*	19/05/2025	1	PZ230210	Pigeon AstVs
25_432	*Columba livia*	19/05/2025	0		
25_433	*Columba livia*	19/05/2025	1	PZ230211	Pigeon AstVs
25_434	*Columba livia*	19/05/2025	0		
25_435	*Columba livia*	26/05/2025	0		
25_436	*Columba livia*	26/05/2025	0		
25_437	*Columba livia*	26/05/2025	0		
25_438	*Columba livia*	26/05/2025	0		
25_470	*Columba livia*	02/06/2025	0		
25_471	*Columba livia*	02/06/2025	0		
25_472	*Columba livia*	02/06/2025	0		
25_473	*Columba livia*	02/06/2025	1	PZ230212	Avastrovirus 2
25_474	*Columba livia*	02/06/2025	0		
25_475	*Columba livia*	02/06/2025	0		
25_476	*Columba livia*	02/06/2025	0		
25_477	*Columba livia*	02/06/2025	0		
25_478	*Columba livia*	02/06/2025	1	PZ230213	Avastrovirus 2
25_479	*Columba livia*	02/06/2025	0		
25_480	*Columba livia*	10/06/2025	0		
25_481	*Columba livia*	10/06/2025	0		
25_482	*Columba livia*	10/06/2025	0		
25_483	*Columba livia*	10/06/2025	0		
25_484	*Columba livia*	10/06/2025	0		
25_485	*Columba livia*	10/06/2025	0		
25_486	*Columba livia*	10/06/2025	0		
25_487	*Columba livia*	19/06/2025	0		
25_488	*Columba livia*	19/06/2025	0		
25_489	*Columba livia*	19/06/2025	1	PZ230214	Pigeon AstVs
25_490	*Columba livia*	19/06/2025	0		
25_491	*Columba livia*	19/06/2025	0		
25_492	*Columba livia*	19/06/2025	1	PZ230215	Pigeon AstVs
25_493	*Columba livia*	19/06/2025	0		
25_494	*Columba livia*	19/06/2025	0		
25_495	*Columba livia*	19/06/2025	0		
25_496	*Columba livia*	19/06/2025	0		
25_497	*Columba livia*	19/06/2025	0		
25_498	*Columba livia*	19/06/2025	0		
25_499	*Columba livia*	19/06/2025	0		
25_500	*Columba livia*	19/06/2025	0		
25_501	*Columba livia*	19/06/2025	0		
25_502	*Columba livia*	19/06/2025	1	PZ230216	Unclassified avian AstVs
25_503	*Columba livia*	07/07/2025	0		
25_504	*Columba livia*	07/07/2025	0		
25_505	*Columba livia*	07/07/2025	0		
25_506	*Columba livia*	07/07/2025	1	PZ230217	Avastrovirus 2
25_507	*Columba livia*	07/07/2025	0		
25_508	*Columba livia*	07/07/2025	0		
25_509	*Columba livia*	15/07/2025	0		
25_510	*Columba livia*	15/07/2025	0		
25_511	*Columba livia*	15/07/2025	0		
25_512	*Columba livia*	15/07/2025	0		
25_513	*Columba livia*	15/07/2025	0		
25_514	*Columba livia*	11/08/2025	1	PZ230218	Pigeon AstVs
25_515	*Columba livia*	11/08/2025	0		
25_516	*Columba livia*	11/08/2025	0		
25_517	*Columba livia*	11/08/2025	1	PZ230219	Pigeon AstVs
25_518	*Columba livia*	11/08/2025	0		
25_519	*Columba livia*	11/08/2025	1	PZ230220	Pigeon AstVs
25_520	*Columba livia*	11/08/2025	0		
25_521	*Columba livia*	11/08/2025	1	PZ230221	Pigeon AstVs
25_522	*Columba livia*	11/08/2025	1	PZ230222	Pigeon AstVs
25_523	*Columba livia*	11/08/2025	1	PZ230223	Pigeon AstVs
25_524	*Columba livia*	11/08/2025	0		
25_525	*Columba livia*	11/08/2025	1	PZ230224	Pigeon AstVs
25_526	*Columba livia*	27/08/2025	0		
25_527	*Columba livia*	27/08/2025	1	PZ230225	Pigeon AstVs
25_528	*Columba livia*	27/08/2025	1	PZ230226	Pigeon AstVs
25_529	*Columba livia*	27/08/2025	0		
25_530	*Columba livia*	27/08/2025	0		
25_531	*Columba livia*	27/08/2025	0		
25_532	*Columba livia*	27/08/2025	0		
25_533	*Columba livia*	27/08/2025	0		
25_534	*Columba livia*	27/08/2025	0		

25_535	*Columba livia*	27/08/2025	1	PZ230227	Pigeon AstVs
25_536	*Columba livia*	27/08/2025	0		
25_537	*Columba livia*	15/09/2025	0		
25_538	*Columba livia*	15/09/2025	0		
25_539	*Columba livia*	15/09/2025	0		
25_540	*Columba livia*	15/09/2025	0		
25_541	*Columba livia*	15/09/2025	0		
25_542	*Columba livia*	15/09/2025	0		
25_543	*Columba livia*	15/09/2025	0		
25_544	*Columba livia*	15/09/2025	0		
25_545	*Columba livia*	15/09/2025	0		
25_546	*Columba livia*	01/10/2025	0		
25_547	*Columba livia*	01/10/2025	0		
25_548	*Columba livia*	01/10/2025	0		
25_549	*Columba livia*	01/10/2025	1	PZ230228	Pigeon AstVs
25_550	*Columba livia*	01/10/2025	1	PZ230229	Pigeon AstVs
25_582	*Columba livia*	16/10/2025	1	PZ230230	Pigeon AstVs
25_583	*Columba livia*	16/10/2025	0		
25_584	*Columba livia*	16/10/2025	1	PZ230231	Pigeon AstVs
25_585	*Columba livia*	16/10/2025	1	PZ230232	Pigeon AstVs
25_586	*Columba livia*	16/10/2025	0		
25_587	*Columba livia*	16/10/2025	0		
25_588	*Columba livia*	16/10/2025	1	PZ230233	Pigeon AstVs
25_589	*Columba livia*	16/10/2025	1	PZ230234	Pigeon AstVs
25_590	*Columba livia*	16/10/2025	0		
25_591	*Columba livia*	16/10/2025	1	PZ230235	Pigeon AstVs
25_592	*Columba livia*	16/10/2025	0		
25_593	*Columba livia*	16/10/2025	0		
25_594	*Columba livia*	29/10/2025	0		
25_595	*Columba livia*	29/10/2025	0		
25_596	*Columba livia*	29/10/2025	1	PZ230236	Pigeon AstVs
25_597	*Columba livia*	29/10/2025	0		
25_648	*Columba livia*	13/11/2025	1	PZ230237	Pigeon AstVs
25_649	*Columba livia*	13/11/2025	0		
25_650	*Columba livia*	13/11/2025	1	PZ230238	Pigeon AstVs
25_651	*Columba livia*	13/11/2025	1	PZ230239	Pigeon AstVs
25_652	*Columba livia*	13/11/2025	0		
25_653	*Columba livia*	13/11/2025	1	PZ230240	Pigeon AstVs
25_654	*Columba livia*	13/11/2025	1	PZ230241	Pigeon AstVs
25_655	*Columba livia*	13/11/2025	0		
25_656	*Columba livia*	13/11/2025	1	PZ230242	Pigeon AstVs
25_657	*Columba livia*	13/11/2025	1	PZ230243	Pigeon AstVs
25_708	*Columba livia*	26/11/2025	1	PZ230244	Pigeon AstVs
25_709	*Columba livia*	26/11/2025	0		
25_710	*Columba livia*	26/11/2025	0		
25_711	*Columba livia*	26/11/2025	0		
25_713	*Columba livia*	26/11/2025	1	PZ230245	Pigeon AstVs
25_714	*Columba livia*	26/11/2025	1	PZ230246	Pigeon AstVs
