# Supplementary material for: Longitudinal detection of astroviruses in feral pigeon droppings reveals a year-round source of viral exposure for endemic bats
Source: J Virol. 2026 Jul 16;100(8):e00657-26. doi: 10.1128/jvi.00657-26 (PMC13483236; doi:10.1128/jvi.00657-26)
Supplement: Supplementary file 2 [file jvi.00657-26-s0002.docx]

**Additional files**

Table S1. Sample information and accession numbers of sequences generated in this study.

Figure S1. Astrovirus detection in feral pigeon droppings across 32 sampling sessions. The stacked bar chart shows the number of collected samples (grey bars) and positive samples (colored segments) for each of the 32 monthly sampling sessions (November 2024–November 2025). Positive samples are colored according to their phylogenetic classification:
